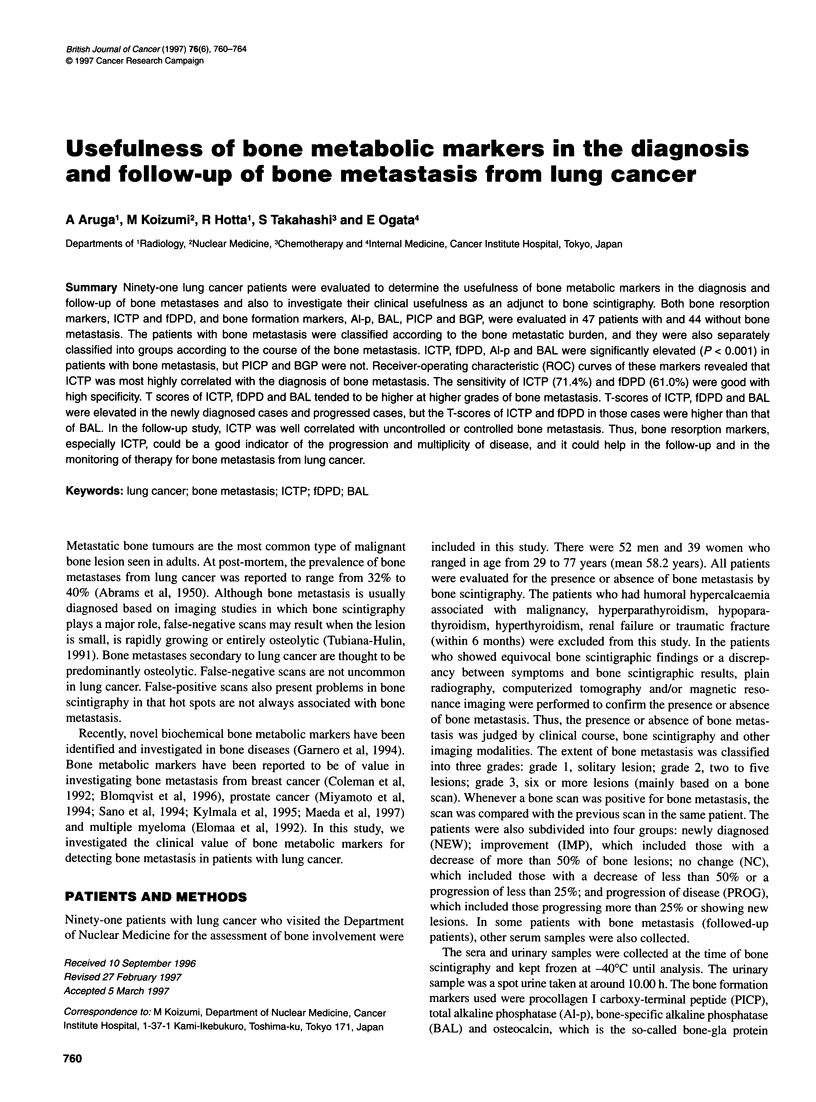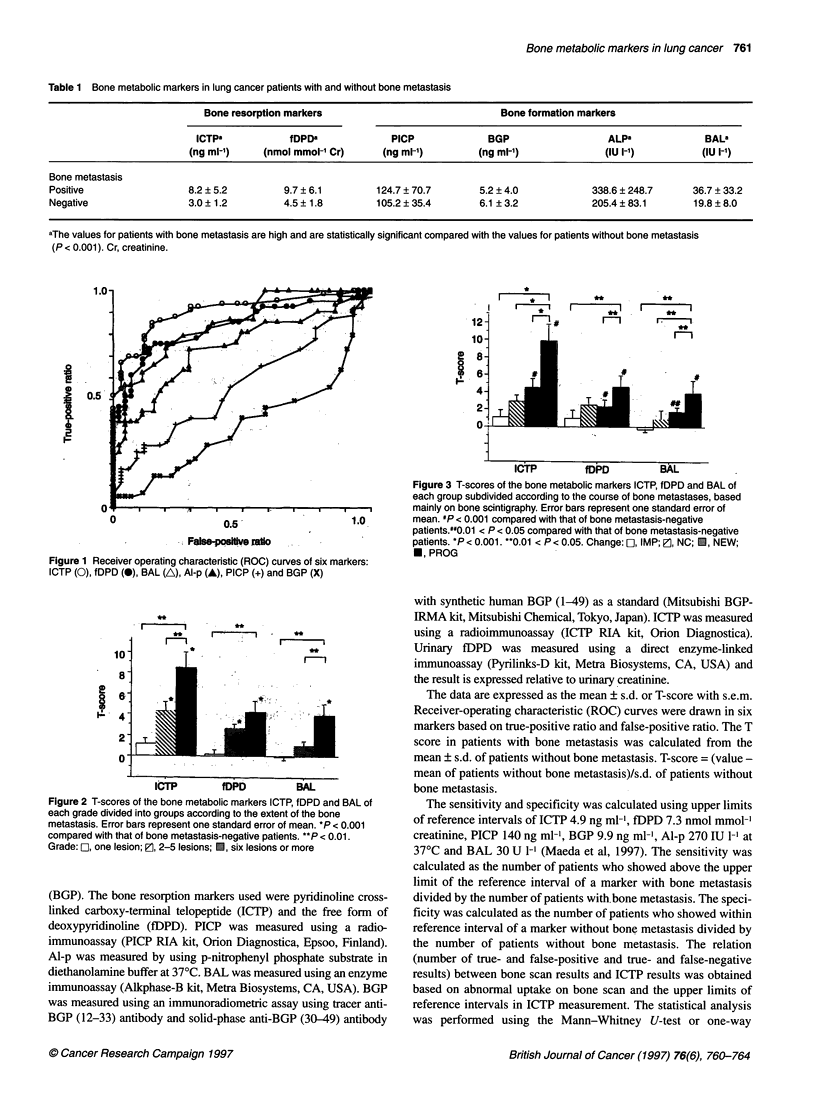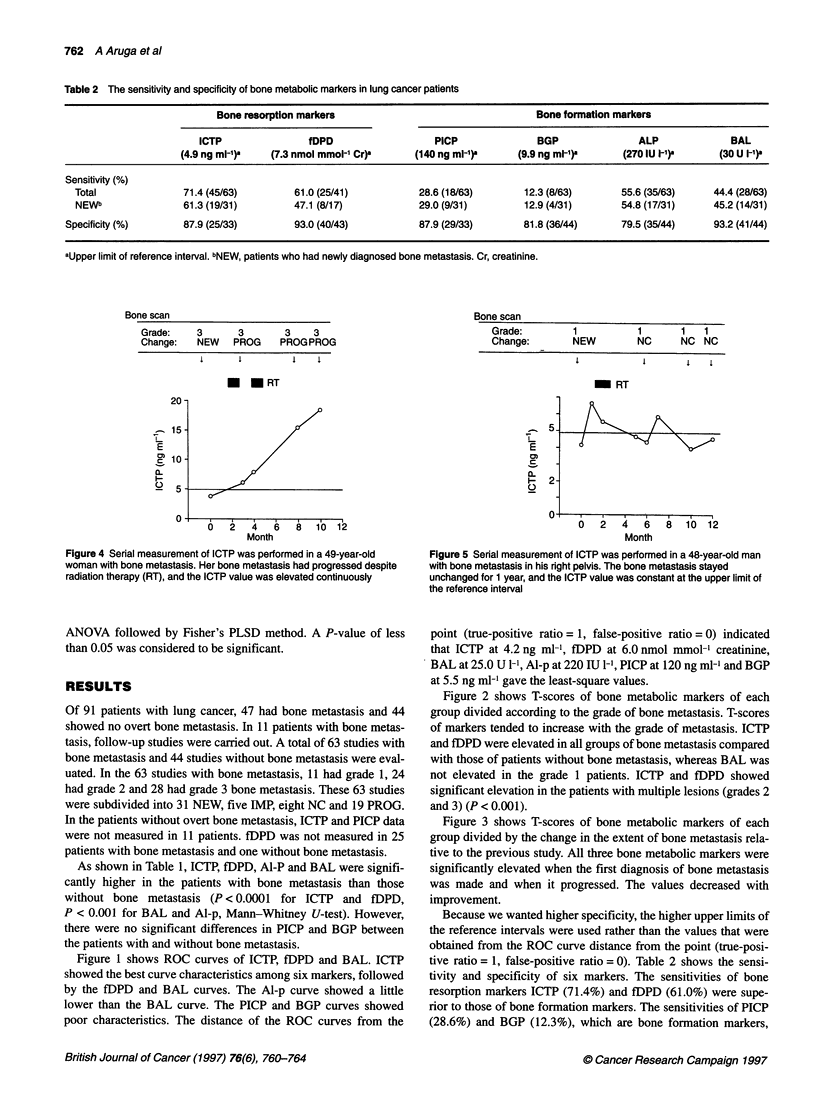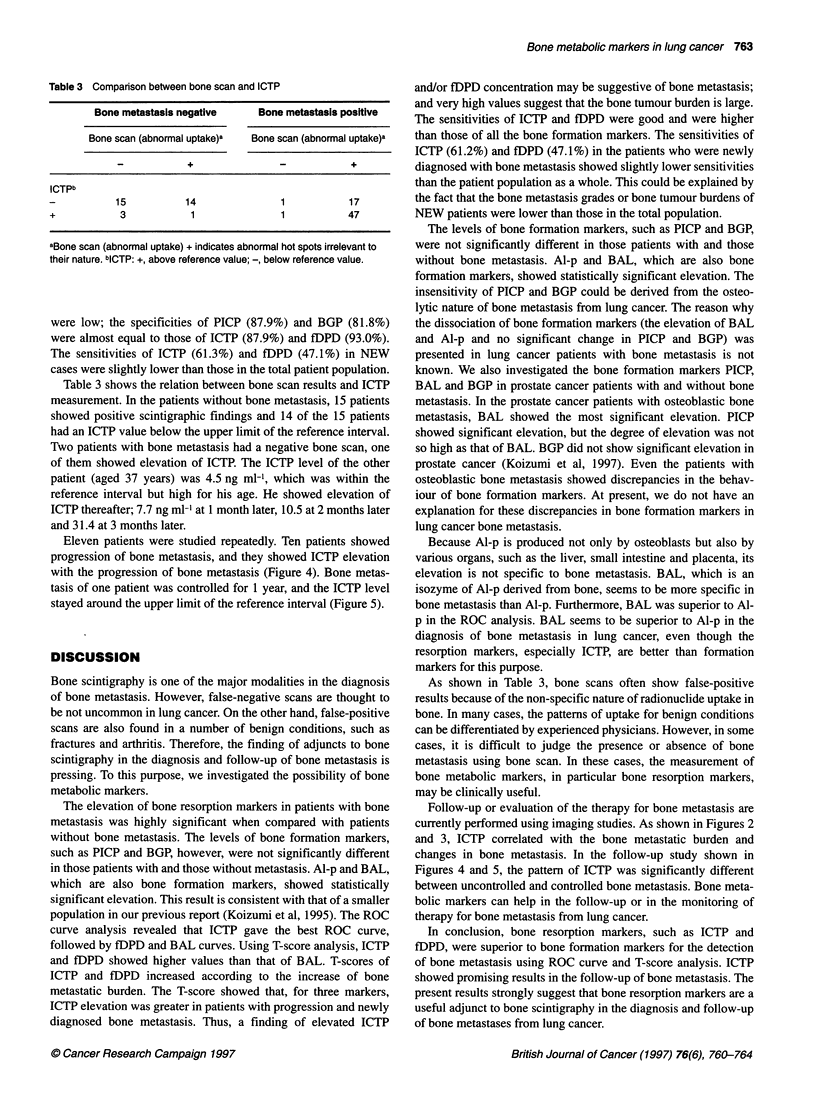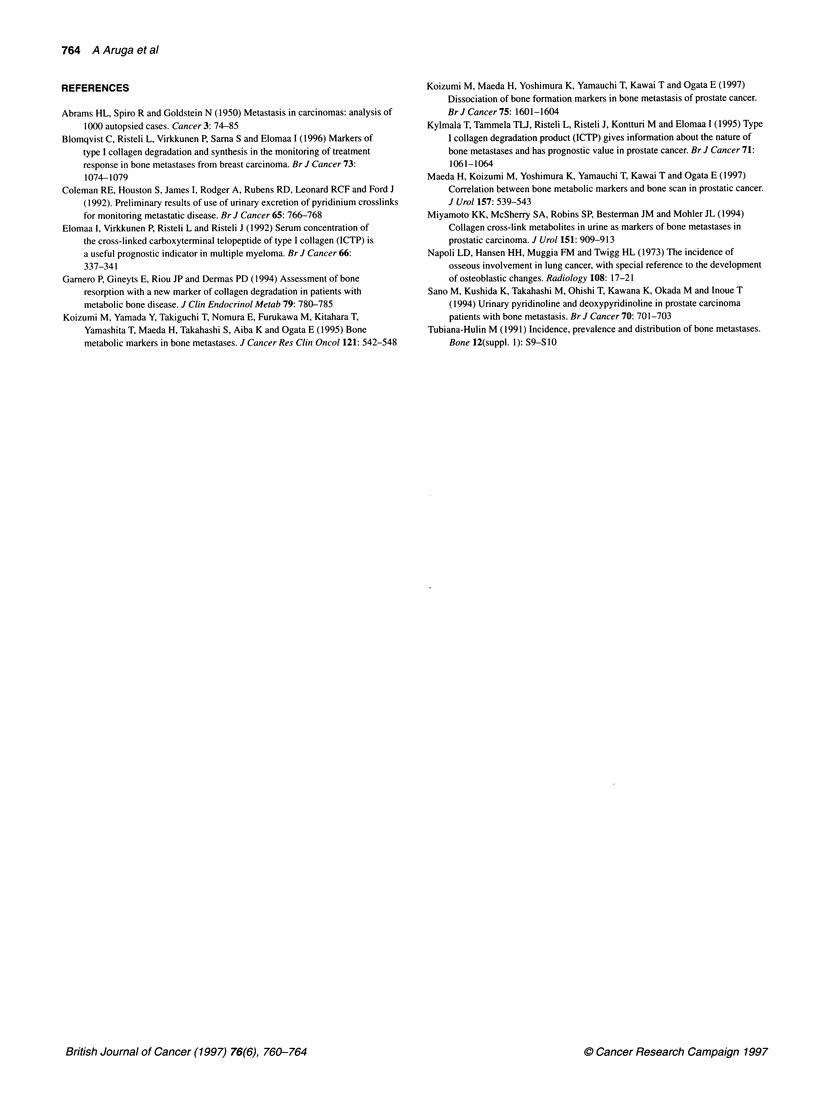# Usefulness of bone metabolic markers in the diagnosis and follow-up of bone metastasis from lung cancer.

**DOI:** 10.1038/bjc.1997.458

**Published:** 1997

**Authors:** A. Aruga, M. Koizumi, R. Hotta, S. Takahashi, E. Ogata

**Affiliations:** Department of Radiology, Cancer Institute Hospital, Tokyo, Japan.

## Abstract

Ninety-one lung cancer patients were evaluated to determine the usefulness of bone metabolic markers in the diagnosis and follow-up of bone metastases and also to investigate their clinical usefulness as an adjunct to bone scintigraphy. Both bone resorption markers, ICTP and fDPD, and bone formation markers, Al-p, BAL, PICP and BGP, were evaluated in 47 patients with and 44 without bone metastasis. The patients with bone metastasis were classified according to the bone metastatic burden, and they were also separately classified into groups according to the course of the bone metastasis. ICTP, fDPD, Al-p and BAL were significantly elevated (P < 0.001) in patients with bone metastasis, but PICP and BGP were not. Receiver-operating characteristic (ROC) curves of these markers revealed that ICTP was most highly correlated with the diagnosis of bone metastasis. The sensitivity of ICTP (71.4%) and fDPD (61.0%) were good with high specificity. T scores of ICTP, fDPD and BAL tended to be higher at higher grades of bone metastasis. T-scores of ICTP, fDPD and BAL were elevated in the newly diagnosed cases and progressed cases, but the T-scores of ICTP and fDPD in those cases were higher than that of BAL. In the follow-up study, ICTP was well correlated with uncontrolled or controlled bone metastasis. Thus, bone resorption markers, especially ICTP, could be a good indicator of the progression and multiplicity of disease, and it could help in the follow-up and in the monitoring of therapy for bone metastasis from lung cancer.


*British Journal of Cancer* (1997) **76**(6), 760–764
© 1997 Cancer Research Campaign

### Usefulness of bone metabolic markers in the diagnosis and follow-up of bone metastasis from lung cancer

**A Aruga[1], M Koizumi[2], R Hotta[1], S Takahashi[3] and E Ogata[4]**

Departments of [1]Radiology, [2]Nuclear Medicine, [3]Chemotherapy and [4]Internal Medicine, Cancer Institute Hospital, Tokyo, Japan

**Summary** Ninety-one lung cancer patients were evaluated to determine the usefulness of bone metabolic markers in the diagnosis and follow-up of bone metastases and also to investigate their clinical usefulness as an adjunct to bone scintigraphy. Both bone resorption markers, ICTP and fDPD, and bone formation markers, Al-p, BAL, PICP and BGP, were evaluated in 47 patients with and 44 without bone metastasis. The patients with bone metastasis were classified according to the bone metastatic burden, and they were also separately classified into groups according to the course of the bone metastasis. ICTP, fDPD, Al-p and BAL were significantly elevated ($P < 0.001$) in patients with bone metastasis, but PICP and BGP were not. Receiver-operating characteristic (ROC) curves of these markers revealed that ICTP was most highly correlated with the diagnosis of bone metastasis. The sensitivity of ICTP (71.4%) and fDPD (61.0%) were good with high specificity. T scores of ICTP, fDPD and BAL tended to be higher at higher grades of bone metastasis. T-scores of ICTP, fDPD and BAL were elevated in the newly diagnosed cases and progressed cases, but the T-scores of ICTP and fDPD in those cases were higher than that of BAL. In the follow-up study, ICTP was well correlated with uncontrolled or controlled bone metastasis. Thus, bone resorption markers, especially ICTP, could be a good indicator of the progression and multiplicity of disease, and it could help in the follow-up and in the monitoring of therapy for bone metastasis from lung cancer.

**Keywords:** lung cancer; bone metastasis; ICTP; fDPD; BAL

Metastatic bone tumours are the most common type of malignant bone lesion seen in adults. At post-mortem, the prevalence of bone metastases from lung cancer was reported to range from 32% to 40% (Abrams et al, 1950). Although bone metastasis is usually diagnosed based on imaging studies in which bone scintigraphy plays a major role, false-negative scans may result when the lesion is small, is rapidly growing or entirely osteolytic (Tubiana-Hulin, 1991). Bone metastases secondary to lung cancer are thought to be predominantly osteolytic. False-negative scans are not uncommon in lung cancer. False-positive scans also present problems in bone scintigraphy in that hot spots are not always associated with bone metastasis.

Recently, novel biochemical bone metabolic markers have been identified and investigated in bone diseases (Garnero et al, 1994). Bone metabolic markers have been reported to be of value in investigating bone metastasis from breast cancer (Coleman et al, 1992; Blomqvist et al, 1996), prostate cancer (Miyamoto et al, 1994; Sano et al, 1994; Kylmala et al, 1995; Maeda et al, 1997) and multiple myeloma (Elomaa et al, 1992). In this study, we investigated the clinical value of bone metabolic markers for detecting bone metastasis in patients with lung cancer.

### PATIENTS AND METHODS

Ninety-one patients with lung cancer who visited the Department of Nuclear Medicine for the assessment of bone involvement were included in this study. There were 52 men and 39 women who ranged in age from 29 to 77 years (mean 58.2 years). All patients were evaluated for the presence or absence of bone metastasis by bone scintigraphy. The patients who had humoral hypercalcaemia associated with malignancy, hyperparathyroidism, hypoparathyroidism, hyperthyroidism, renal failure or traumatic fracture (within 6 months) were excluded from this study. In the patients who showed equivocal bone scintigraphic findings or a discrepancy between symptoms and bone scintigraphic results, plain radiography, computerized tomography and/or magnetic resonance imaging were performed to confirm the presence or absence of bone metastasis. Thus, the presence or absence of bone metastasis was judged by clinical course, bone scintigraphy and other imaging modalities. The extent of bone metastasis was classified into three grades: grade 1, solitary lesion; grade 2, two to five lesions; grade 3, six or more lesions (mainly based on a bone scan). Whenever a bone scan was positive for bone metastasis, the scan was compared with the previous scan in the same patient. The patients were also subdivided into four groups: newly diagnosed (NEW); improvement (IMP), which included those with a decrease of more than 50% of bone lesions; no change (NC), which included those with a decrease of less than 50% or a progression of less than 25%; and progression of disease (PROG), which included those progressing more than 25% or showing new lesions. In some patients with bone metastasis (followed-up patients), other serum samples were also collected.

The sera and urinary samples were collected at the time of bone scintigraphy and kept frozen at –40°C until analysis. The urinary sample was a spot urine taken at around 10.00 h. The bone formation markers used were procollagen I carboxy-terminal peptide (PICP), total alkaline phosphatase (Al-p), bone-specific alkaline phosphatase (BAL) and osteocalcin, which is the so-called bone-gla protein

*Received 10 September 1996*
*Revised 27 February 1997*
*Accepted 5 March 1997*

*Correspondence to:* M Koizumi, Department of Nuclear Medicine, Cancer Institute Hospital, 1-37-1 Kami-Ikebukuro, Toshima-ku, Tokyo 171, Japan

**Table 1** Bone metabolic markers in lung cancer patients with and without bone metastasis

| | Bone resorption markers | | Bone formation markers | | | |
|---|---|---|---|---|---|---|
| | ICTP[a] (ng ml$^{-1}$) | fDPD[a] (nmol mmol$^{-1}$ Cr) | PICP (ng ml$^{-1}$) | BGP (ng ml$^{-1}$) | ALP[a] (IU l$^{-1}$) | BAL[a] (IU l$^{-1}$) |
| Bone metastasis | | | | | | |
| Positive | 8.2 ± 5.2 | 9.7 ± 6.1 | 124.7 ± 70.7 | 5.2 ± 4.0 | 338.6 ± 248.7 | 36.7 ± 33.2 |
| Negative | 3.0 ± 1.2 | 4.5 ± 1.8 | 105.2 ± 35.4 | 6.1 ± 3.2 | 205.4 ± 83.1 | 19.8 ± 8.0 |

[a]The values for patients with bone metastasis are high and are statistically significant compared with the values for patients without bone metastasis ($P < 0.001$). Cr, creatinine.

**Figure 1** Receiver operating characteristic (ROC) curves of six markers: ICTP (○), fDPD (●), BAL (△), Al-p (▲), PICP (+) and BGP (X)

**Figure 2** T-scores of the bone metabolic markers ICTP, fDPD and BAL of each grade divided into groups according to the extent of the bone metastasis. Error bars represent one standard error of mean. *$P < 0.001$ compared with that of bone metastasis-negative patients. **$P < 0.01$. Grade: □, one lesion; ▨, 2–5 lesions; ▩, six lesions or more

**Figure 3** T-scores of the bone metabolic markers ICTP, fDPD and BAL of each group subdivided according to the course of bone metastases, based mainly on bone scintigraphy. Error bars represent one standard error of mean. [#]$P < 0.001$ compared with that of bone metastasis-negative patients.[##]$0.01 < P < 0.05$ compared with that of bone metastasis-negative patients. *$P < 0.001$. **$0.01 < P < 0.05$. Change: □, IMP; ▨, NC; ▩, NEW; ■, PROG

(BGP). The bone resorption markers used were pyridinoline cross-linked carboxy-terminal telopeptide (ICTP) and the free form of deoxypyridinoline (fDPD). PICP was measured using a radioimmunoassay (PICP RIA kit, Orion Diagnostica, Epsoo, Finland). Al-p was measured by using p-nitrophenyl phosphate substrate in diethanolamine buffer at 37°C. BAL was measured using an enzyme immunoassay (Alkphase-B kit, Metra Biosystems, CA, USA). BGP was measured using an immunoradiometric assay using tracer anti-BGP (12–33) antibody and solid-phase anti-BGP (30–49) antibody with synthetic human BGP (1–49) as a standard (Mitsubishi BGP-IRMA kit, Mitsubishi Chemical, Tokyo, Japan). ICTP was measured using a radioimmunoassay (ICTP RIA kit, Orion Diagnostica). Urinary fDPD was measured using a direct enzyme-linked immunoassay (Pyrilinks-D kit, Metra Biosystems, CA, USA) and the result is expressed relative to urinary creatinine.

The data are expressed as the mean ± s.d. or T-score with s.e.m. Receiver-operating characteristic (ROC) curves were drawn in six markers based on true-positive ratio and false-positive ratio. The T score in patients with bone metastasis was calculated from the mean ± s.d. of patients without bone metastasis. T-score = (value – mean of patients without bone metastasis)/s.d. of patients without bone metastasis.

The sensitivity and specificity was calculated using upper limits of reference intervals of ICTP 4.9 ng ml$^{-1}$, fDPD 7.3 nmol mmol$^{-1}$ creatinine, PICP 140 ng ml$^{-1}$, BGP 9.9 ng ml$^{-1}$, Al-p 270 IU l$^{-1}$ at 37°C and BAL 30 U l$^{-1}$ (Maeda et al, 1997). The sensitivity was calculated as the number of patients who showed above the upper limit of the reference interval of a marker with bone metastasis divided by the number of patients with bone metastasis. The specificity was calculated as the number of patients who showed within reference interval of a marker without bone metastasis divided by the number of patients without bone metastasis. The relation (number of true- and false-positive and true- and false-negative results) between bone scan results and ICTP results was obtained based on abnormal uptake on bone scan and the upper limits of reference intervals in ICTP measurement. The statistical analysis was performed using the Mann–Whitney $U$-test or one-way

Table 2 The sensitivity and specificity of bone metabolic markers in lung cancer patients

| | Bone resorption markers | | Bone formation markers | | | |
|---|---|---|---|---|---|---|
| | ICTP (4.9 ng $ml^{-1}$)[a] | fDPD (7.3 nmol $mmol^{-1}$ Cr)[a] | PICP (140 ng $ml^{-1}$)[a] | BGP (9.9 ng $ml^{-1}$)[a] | ALP (270 IU $l^{-1}$)[a] | BAL (30 U $l^{-1}$)[a] |
| Sensitivity (%) | | | | | | |
| Total | 71.4 (45/63) | 61.0 (25/41) | 28.6 (18/63) | 12.3 (8/63) | 55.6 (35/63) | 44.4 (28/63) |
| NEW[b] | 61.3 (19/31) | 47.1 (8/17) | 29.0 (9/31) | 12.9 (4/31) | 54.8 (17/31) | 45.2 (14/31) |
| Specificity (%) | 87.9 (25/33) | 93.0 (40/43) | 87.9 (29/33) | 81.8 (36/44) | 79.5 (35/44) | 93.2 (41/44) |

[a]Upper limit of reference interval. [b]NEW, patients who had newly diagnosed bone metastasis. Cr, creatinine.

Figure 4 Serial measurement of ICTP was performed in a 49-year-old woman with bone metastasis. Her bone metastasis had progressed despite radiation therapy (RT), and the ICTP value was elevated continuously

Figure 5 Serial measurement of ICTP was performed in a 48-year-old man with bone metastasis in his right pelvis. The bone metastasis stayed unchanged for 1 year, and the ICTP value was constant at the upper limit of the reference interval

ANOVA followed by Fisher's PLSD method. A $P$-value of less than 0.05 was considered to be significant.

### RESULTS

Of 91 patients with lung cancer, 47 had bone metastasis and 44 showed no overt bone metastasis. In 11 patients with bone metastasis, follow-up studies were carried out. A total of 63 studies with bone metastasis and 44 studies without bone metastasis were evaluated. In the 63 studies with bone metastasis, 11 had grade 1, 24 had grade 2 and 28 had grade 3 bone metastasis. These 63 studies were subdivided into 31 NEW, five IMP, eight NC and 19 PROG. In the patients without overt bone metastasis, ICTP and PICP data were not measured in 11 patients. fDPD was not measured in 25 patients with bone metastasis and one without bone metastasis.

As shown in Table 1, ICTP, fDPD, Al-P and BAL were significantly higher in the patients with bone metastasis than those without bone metastasis ($P < 0.0001$ for ICTP and fDPD, $P < 0.001$ for BAL and Al-p, Mann–Whitney $U$-test). However, there were no significant differences in PICP and BGP between the patients with and without bone metastasis.

Figure 1 shows ROC curves of ICTP, fDPD and BAL. ICTP showed the best curve characteristics among six markers, followed by the fDPD and BAL curves. The Al-p curve showed a little lower than the BAL curve. The PICP and BGP curves showed poor characteristics. The distance of the ROC curves from the point (true-positive ratio = 1, false-positive ratio = 0) indicated that ICTP at 4.2 ng $ml^{-1}$, fDPD at 6.0 nmol $mmol^{-1}$ creatinine, BAL at 25.0 U $l^{-1}$, Al-p at 220 IU $l^{-1}$, PICP at 120 ng $ml^{-1}$ and BGP at 5.5 ng $ml^{-1}$ gave the least-square values.

Figure 2 shows T-scores of bone metabolic markers of each group divided according to the grade of bone metastasis. T-scores of markers tended to increase with the grade of metastasis. ICTP and fDPD were elevated in all groups of bone metastasis compared with those of patients without bone metastasis, whereas BAL was not elevated in the grade 1 patients. ICTP and fDPD showed significant elevation in the patients with multiple lesions (grades 2 and 3) ($P < 0.001$).

Figure 3 shows T-scores of bone metabolic markers of each group divided by the change in the extent of bone metastasis relative to the previous study. All three bone metabolic markers were significantly elevated when the first diagnosis of bone metastasis was made and when it progressed. The values decreased with improvement.

Because we wanted higher specificity, the higher upper limits of the reference intervals were used rather than the values that were obtained from the ROC curve distance from the point (true-positive ratio = 1, false-positive ratio = 0). Table 2 shows the sensitivity and specificity of six markers. The sensitivities of bone resorption markers ICTP (71.4%) and fDPD (61.0%) were superior to those of bone formation markers. The sensitivities of PICP (28.6%) and BGP (12.3%), which are bone formation markers,

© Cancer Research Campaign 1997

**Table 3** Comparison between bone scan and ICTP

| | Bone metastasis negative | | Bone metastasis positive | |
|---|---|---|---|---|
| | Bone scan (abnormal uptake)[a] | | Bone scan (abnormal uptake)[a] | |
| | – | + | – | + |
| ICTP[b] | | | | |
| – | 15 | 14 | 1 | 17 |
| + | 3 | 1 | 1 | 47 |

[a]Bone scan (abnormal uptake) + indicates abnormal hot spots irrelevant to their nature. [b]ICTP: +, above reference value; –, below reference value.

were low; the specificities of PICP (87.9%) and BGP (81.8%) were almost equal to those of ICTP (87.9%) and fDPD (93.0%). The sensitivities of ICTP (61.3%) and fDPD (47.1%) in NEW cases were slightly lower than those in the total patient population.

Table 3 shows the relation between bone scan results and ICTP measurement. In the patients without bone metastasis, 15 patients showed positive scintigraphic findings and 14 of the 15 patients had an ICTP value below the upper limit of the reference interval. Two patients with bone metastasis had a negative bone scan, one of them showed elevation of ICTP. The ICTP level of the other patient (aged 37 years) was 4.5 ng $ml^{-1}$, which was within the reference interval but high for his age. He showed elevation of ICTP thereafter; 7.7 ng $ml^{-1}$ at 1 month later, 10.5 at 2 months later and 31.4 at 3 months later.

Eleven patients were studied repeatedly. Ten patients showed progression of bone metastasis, and they showed ICTP elevation with the progression of bone metastasis (Figure 4). Bone metastasis of one patient was controlled for 1 year, and the ICTP level stayed around the upper limit of the reference interval (Figure 5).

### DISCUSSION

Bone scintigraphy is one of the major modalities in the diagnosis of bone metastasis. However, false-negative scans are thought to be not uncommon in lung cancer. On the other hand, false-positive scans are also found in a number of benign conditions, such as fractures and arthritis. Therefore, the finding of adjuncts to bone scintigraphy in the diagnosis and follow-up of bone metastasis is pressing. To this purpose, we investigated the possibility of bone metabolic markers.

The elevation of bone resorption markers in patients with bone metastasis was highly significant when compared with patients without bone metastasis. The levels of bone formation markers, such as PICP and BGP, however, were not significantly different in those patients with and those without metastasis. Al-p and BAL, which are also bone formation markers, showed statistically significant elevation. This result is consistent with that of a smaller population in our previous report (Koizumi et al, 1995). The ROC curve analysis revealed that ICTP gave the best ROC curve, followed by fDPD and BAL curves. Using T-score analysis, ICTP and fDPD showed higher values than that of BAL. T-scores of ICTP and fDPD increased according to the increase of bone metastatic burden. The T-score showed that, for three markers, ICTP elevation was greater in patients with progression and newly diagnosed bone metastasis. Thus, a finding of elevated ICTP and/or fDPD concentration may be suggestive of bone metastasis; and very high values suggest that the bone tumour burden is large. The sensitivities of ICTP and fDPD were good and were higher than those of all the bone formation markers. The sensitivities of ICTP (61.2%) and fDPD (47.1%) in the patients who were newly diagnosed with bone metastasis showed slightly lower sensitivities than the patient population as a whole. This could be explained by the fact that the bone metastasis grades or bone tumour burdens of NEW patients were lower than those in the total population.

The levels of bone formation markers, such as PICP and BGP, were not significantly different in those patients with and those without bone metastasis. Al-p and BAL, which are also bone formation markers, showed statistically significant elevation. The insensitivity of PICP and BGP could be derived from the osteolytic nature of bone metastasis from lung cancer. The reason why the dissociation of bone formation markers (the elevation of BAL and Al-p and no significant change in PICP and BGP) was presented in lung cancer patients with bone metastasis is not known. We also investigated the bone formation markers PICP, BAL and BGP in prostate cancer patients with and without bone metastasis. In the prostate cancer patients with osteoblastic bone metastasis, BAL showed the most significant elevation. PICP showed significant elevation, but the degree of elevation was not so high as that of BAL. BGP did not show significant elevation in prostate cancer (Koizumi et al, 1997). Even the patients with osteoblastic bone metastasis showed discrepancies in the behaviour of bone formation markers. At present, we do not have an explanation for these discrepancies in bone formation markers in lung cancer bone metastasis.

Because Al-p is produced not only by osteoblasts but also by various organs, such as the liver, small intestine and placenta, its elevation is not specific to bone metastasis. BAL, which is an isozyme of Al-p derived from bone, seems to be more specific in bone metastasis than Al-p. Furthermore, BAL was superior to Al-p in the ROC analysis. BAL seems to be superior to Al-p in the diagnosis of bone metastasis in lung cancer, even though the resorption markers, especially ICTP, are better than formation markers for this purpose.

As shown in Table 3, bone scans often show false-positive results because of the non-specific nature of radionuclide uptake in bone. In many cases, the patterns of uptake for benign conditions can be differentiated by experienced physicians. However, in some cases, it is difficult to judge the presence or absence of bone metastasis using bone scan. In these cases, the measurement of bone metabolic markers, in particular bone resorption markers, may be clinically useful.

Follow-up or evaluation of the therapy for bone metastasis are currently performed using imaging studies. As shown in Figures 2 and 3, ICTP correlated with the bone metastatic burden and changes in bone metastasis. In the follow-up study shown in Figures 4 and 5, the pattern of ICTP was significantly different between uncontrolled and controlled bone metastasis. Bone metabolic markers can help in the follow-up or in the monitoring of therapy for bone metastasis from lung cancer.

In conclusion, bone resorption markers, such as ICTP and fDPD, were superior to bone formation markers for the detection of bone metastasis using ROC curve and T-score analysis. ICTP showed promising results in the follow-up of bone metastasis. The present results strongly suggest that bone resorption markers are a useful adjunct to bone scintigraphy in the diagnosis and follow-up of bone metastases from lung cancer.

© Cancer Research Campaign 1997

### REFERENCES

Abrams HL, Spiro R and Goldstein N (1950) Metastasis in carcinomas: analysis of 1000 autopsied cases. *Cancer* **3**: 74–85

Blomqvist C, Risteli L, Virkkunen P, Sarna S and Elomaa I (1996) Markers of type I collagen degradation and synthesis in the monitoring of treatment response in bone metastases from breast carcinoma. *Br J Cancer* **73**: 1074–1079

Coleman RE, Houston S, James I, Rodger A, Rubens RD, Leonard RCF and Ford J (1992). Preliminary results of use of urinary excretion of pyridinium crosslinks for monitoring metastatic disease. *Br J Cancer* **65**: 766–768

Elomaa I, Virkkunen P, Risteli L and Risteli J (1992) Serum concentration of the cross-linked carboxyterminal telopeptide of type I collagen (ICTP) is a useful prognostic indicator in multiple myeloma. *Br J Cancer* **66**: 337–341

Garnero P, Gineyts E, Riou JP and Dermas PD (1994) Assessment of bone resorption with a new marker of collagen degradation in patients with metabolic bone disease. *J Clin Endocrinol Metab* **79**: 780–785

Koizumi M, Yamada Y, Takiguchi T, Nomura E, Furukawa M, Kitahara T, Yamashita T, Maeda H, Takahashi S, Aiba K and Ogata E (1995) Bone metabolic markers in bone metastases. *J Cancer Res Clin Oncol* **121**: 542–548

Koizumi M, Maeda H, Yoshimura K, Yamauchi T, Kawai T and Ogata E (1997) Dissociation of bone formation markers in bone metastasis of prostate cancer. *Br J Cancer* **75**: 1601–1604

Kylmala T, Tammela TLJ, Risteli L, Risteli J, Kontturi M and Elomaa I (1995) Type I collagen degradation product (ICTP) gives information about the nature of bone metastases and has prognostic value in prostate cancer. *Br J Cancer* **71**: 1061–1064

Maeda H, Koizumi M, Yoshimura K, Yamauchi T, Kawai T and Ogata E (1997) Correlation between bone metabolic markers and bone scan in prostatic cancer. *J Urol* **157**: 539–543

Miyamoto KK, McSherry SA, Robins SP, Besterman JM and Mohler JL (1994) Collagen cross-link metabolites in urine as markers of bone metastases in prostatic carcinoma. *J Urol* **151**: 909–913

Napoli LD, Hansen HH, Muggia FM and Twigg HL (1973) The incidence of osseous involvement in lung cancer, with special reference to the development of osteoblastic changes. *Radiology* **108**: 17–21

Sano M, Kushida K, Takahashi M, Ohishi T, Kawana K, Okada M and Inoue T (1994) Urinary pyridinoline and deoxypyridinoline in prostate carcinoma patients with bone metastasis. *Br J Cancer* **70**: 701–703

Tubiana-Hulin M (1991) Incidence, prevalence and distribution of bone metastases. *Bone* **12**(suppl. 1): S9–S10

*© Cancer Research Campaign 1997*